# Rheumatic Fever Prevention Program: Long-Term Evolution and Outcomes

**DOI:** 10.3389/fped.2014.00141

**Published:** 2015-01-06

**Authors:** Cleonice Carvalho Coelho Mota, Zilda Maria Alves Meira, Rosangela Nicoli Graciano, Fernando Felipe Graciano, Fátima Derlene Rocha Araújo

**Affiliations:** ^1^Department of Pediatrics, Hospital das Clínicas da UFMG, Federal University of Minas Gerais (UFMG), Belo Horizonte, Brazil; ^2^Hospital das Clínicas da UFMG, Belo Horizonte, Brazil

**Keywords:** rheumatic fever, rheumatic heart disease, secondary prophylaxis, recurrences, valvar dysfunction

## Abstract

This investigation aims to analyze the profile of long-term evolution of rheumatic fever in children and adolescents and outcomes after the control of recurrences. The cohort involved 702 patients followed from 1.3 to 16.9 years covering the two periods, before and after the implementation of a prevention program. Besides the establishment of the Reference Center in the State of Minas Gerais and the implementation of strategies to promote the compliance to prophylaxis, a project for education of health professionals was carried out in 23 cities. In addition to the clinical and epidemiological profile, the severity of the disease was analyzed. Mixed lesions were found in 27.1%, valvar regurgitation in 72.9%, and complete regression of the valvar lesions was seen in 34.4% of the patients, mostly presenting mild dysfunctions. The recurrence rate per patient-year was 0.058 and out of a total of 85 recurrences, 21.4% occurred in the first and 7.5% in the second period. More severe degrees of carditis and significant valvar sequels presented a higher prevalence in patients with recurrences. The comparative analysis between the two periods showed no changes regarding the age at the primary attack, gender, type, and site of valvar lesions and affected joints; however, important modifications in the indices of severity were observed after the control of recurrences. A significant decrease in the prevalence of severe carditis, obstructive valvar sequels, hospital admissions, surgical approach, and deaths was seen. This investigation showed that although the clinical profile of presentation remains unchanged, the control of repeated attacks can improve the morbimortality rates. In this context, the secondary prophylaxis should be the first priority in the control of the disease in developing countries, taking into account the difficulties found for effective primordial and primary prevention.

## Introduction

Rheumatic fever (RF) and its more serious consequence, the rheumatic heart disease (RHD), continue to pose a significant problem in several parts of the world. Although the incidence of the disease in developed areas have markedly declined after the 1950s, RHD remains the leading cause of acquired cardiac disease in developing countries among patients aged from 5 to 30 years (1, 2). On a global scale, and considering all age groups, an estimated 15.6–19.6 million people suffer from RHD (3). As a result of a disability acquired at an early age, valvar sequels can be carried throughout life and account for increased rates of hospitalization, surgery, and death. In this setting, an estimated 6.6 million disability-adjusted life years are lost yearly to the disease worldwide (4). The socioeconomic factors involving considerable personal and collective costs constitute important determinants of the disease burden. Maternal low education level has been associated with recurrent attacks and identified as an independent risk factor for significant chronic valvar disease (5). Environmental factors, mainly poor housing and overcrowding, must also be included in this context (1, 4).

More recently, large-scale echocardiographic screenings have shown a prevalence of RHD much higher than previously thought, ranging from 5 to 10-fold when compared to auscultation (6–13). It is a fact that some patients develop RHD, but the clinical history of previous acute episodes can go unnoticed due to poor access to health services or the presence of mild or subclinical valvar lesions. This scenario of underdiagnosis is worrying as without prevention of recurrences, the risks of more advanced disease are considerable. On the other hand, the overdiagnosis results in the undesirable exposure of non-rheumatic patients to the rigors of prophylaxis. Thus, an accurate diagnosis is essential to make adequate decisions in both directions. The international echocardiographic criteria for the diagnosis of RHD, recently established by the World Heart Federation, have given a very important contribution to diagnostic accuracy, mainly to the identification of patients without a past history of RF (14).

The magnitude of the valvular sequels has been associated with the degree of cardiac involvement during the acute phase (15–17). Additionally, as it has long been noticed, the valve deformity can be progressive even in the absence of recurrences as a result from the process of cicatrization (18, 19). Nonetheless, repeated acute attacks play an important role in the unfavorable evolution and severity of RHD. Considering this potential evolution, it is a fact that there is no curative therapy. After the establishment of valvular damage, any treatment, clinical or surgical, is palliative (20). Faced with our limitations in the therapeutic approach of patients with RF and RHD, continuing attention must be given to the primary and secondary prophylaxis. The prompt introduction of an effective secondary prophylaxis can avoid the worsening of preexisting valvar dysfunctions or the appearance of new lesions related to the subsequent acute episodes. In areas where the disease is prevalent, control programs require investments. Strategies are needed to implement the adherence of patients to the regimes of secondary prophylaxis to avoid recurrences.

This investigation aims to analyze the profile of long-term evolution of patients with rheumatic valvar lesions and the outcomes after the control of recurrences, which followed the implementation of the Prevention Program for Rheumatic Fever-UFMG.

## Materials and Methods

### Study population and data collection

This investigation enrolled 702 children and adolescents with diagnosis of RF based on the revised Jones criteria (21). The patients were consecutively attended from July 1977 to February 2000 at the Division of Pediatric Cardiology/Hospital das Clínicas-UFMG and were followed by the same team of pediatric cardiologists over a period ranging from 1.3 to 16.9 years (mean = 6.8 ± 2.6 years). The age at admission ranged from 2.6 to 19.9 years (mean: 10.3 ± 3.1 years; median 9.8 years).

Outpatients and inpatients databases, records, and referring reports from the local health centers were retrospectively investigated for all patients with the diagnosis of RF, acute or chronic phase. Information was sought in the past history, first clinical evaluation, and follow-up, besides electrocardiogram, chest roentgenogram, and echocardiogram records.

### Program design and strategies

The patients were referred during the acute or chronic phases of the disease to the Division of Pediatric Cardiology-UFMG which, in 1986, established the Prevention Program for Rheumatic Fever-UFMG. Since 1988, the university hospital has been the Reference Center in Minas Gerais, a Brazilian state with 0.59 million km^2^, 853 municipalities, and 19.6 million inhabitants. As part of the program, health professional education was carried out in the capital and other 22 cities of the state, grouped by regions and supported by local coordinators. The initiative also included distribution of educational material, promotion of scientific events, and was preceded by divulgation in the printed and electronic media.

The activities of the program were integrated into the existing public health infrastructure, and the following strategies have been adopted to assist patients at the Reference Center for Rheumatic Fever since 1988: the establishment of the Rheumatic Fever Outpatients Clinic at the university hospital; introduction of protocols for standardized approach to diagnosis and management; medical care and orientation provided by a multidisciplinary team; special attention to promote health education for patients and families; appointments for first visits demanded by the public healthcare state system were centralized and organized by the Health Secretary; transportation for patients and parents provided; routine clinical follow-up no longer than at every 6 months; besides the laboratorial exams demanded by clinical evaluation, routine investigation of ASO titer at 6 months’ interval for all patients; echocardiographic screening for all definitive or suspected cases of RF or RHD, even those without clinical evidence of cardiac damage; reporting back to referring health centers to support the local follow-up of patients; distribution of free medication; secondary prophylaxis regimens with intramuscular benzathine penicillin injections at 21 days’ interval; introduction of a prophylaxis card to control the compliance; active search for missing patients and accommodation at the university hospital for inpatients’ parents.

### Data analysis

Demographical, clinical, and epidemiological data were assembled and patients were divided into two groups according to the period of admission: before and after the establishment of the program, from July 1977 to July 1988 and from August 1988 to February 2000, respectively. The degree of carditis and valvular sequels was classified as mild, moderate, and severe according to the established criteria on the protocol for medical assistance (18). Moderate to severe and severe degrees of cardiac involvement for both acute and chronic phases were classified as significant. The characterization of an established RHD was defined at least 1 year after the acute phase. The last clinical and laboratorial evaluations in each respective period of investigation were considered for the characterization of the disease. Variables were summarized as percentage, and categorical variables were compared using Fisher exact test or Pearson’s chi-square test. When *p*-value <0.05 was reported, results were identified as significant.

### Institutional board approval

This project was approved by the Ethics Committee on Human Research-Federal University of Minas Gerais, Belo Horizonte, Brazil. Written informed consent was obtained from the parents of patients enrolled in the study.

## Results

The mean age at the first acute episode was 9.1 ± 4.1 years. The distribution patients by gender was similar (F: 50.4% M: 49.6%), except for those with Sydenham chorea (F/M = 1.6; *p*: 0.0013). In the distribution of patients according to the Jones criteria, carditis (66.2%) and polyarthritis (70.4%), isolated or combined, were the major manifestations most commonly observed.

As regards the type and site of valvular lesions in the chronic phase, valvular regurgitation was present in 72.9% and mixed lesions in 27.1% of patients. The mitral valve was the most affected. Isolated mitral dysfunction or in association with other valvar lesions were seen in 98.2% of patients and almost a half of patients (44.2%) presented isolated mitral regurgitation. Aortic regurgitation was found in 37.8% and most often seen concurrently with an affected mitral valve. No patients with aortic stenosis or isolated mitral stenosis were seen. Complete regression of the valvular lesions at echocardiographic interrogation was observed in 34.4% of the patients, mostly presenting a mild dysfunction. Carditis was diagnosed in 465 patients and classified as severe in 39.8%, moderate in 28.6%, and mild in 31.6%.

According to the past history and before being admitted to the hospital, 34.6% of children and adolescents had presented recurrences, 22.2% had one, and 12.4% had two or more subsequent episodes. After admission, the recurrence rate per patient-year was 0.058, which means that out of a total of 85 recurrent episodes, 51/238 (21.4%) occurred during the first period of investigation and 34/454 (7.5%) during the second one.

When the entire patient population was considered, a higher prevalence of significant carditis (*p*: 0.000) and more severe valvar sequels (*p*: 0.000) were seen in patients with recurrences (Tables 1 and 2).

**Table 1:** **Prevalence of significant carditis at primary episodes and recurrences**.

Significant degree of carditis	Acute episodes		Total of episodes, *n* (%)	*p*	OR	95% CI
	Primary *n* (%)	Recurrences *n* (%)				
Yes	141 (30.3)	69 (57.1)	210 (35.9)	0.000	3.093	1.727– 5.540
No	324 (69.7)	51 (42.9)	375 (64.1)			
Total *n* (%)	465 (100)	120 (100)	585 (100)			

**Table 2:** **Prevalence of significant valvular sequels according to the presence of recurrences**.

Significant valvular sequels	Recurrences		Total patients, *n* (%)	*p*	OR	95% CI
	Yes, *n* (%)	No, *n* (%)	
Yes	98 (40.4)	47 (10.2)	145 (20.2)	0.000	3.000	1.697– 5.302
No	144 (59.7)	413 (89.8)	557 (79.4)			
Total *n* (%)	242 (100)	460 (100)	702 (100)			

The comparative analysis of the two periods of investigation showed that the clinical profile of the acute phase, first episodes or subsequent attacks, was unchanged regarding the age at the primary attack, gender, frequency, and site of affected joints, besides the most frequent association of valvular lesions (Table 3). However, after the decrease in the frequency of recurrences, which followed the improvement of the delivery and adherence to secondary prophylaxis, important modifications in the severity of the disease were observed during the follow-up. A decrease in the frequency of severe carditis, stenotic valvular lesions, hospital admissions, surgical approach, and deaths was noticed showing significant differences in all indices between the two periods of investigation (Table 4).

**Table 3:** **Comparative analysis of the clinical profile of presentation**.

Clinical profile	Time interval		*p*	OR	CI
	07/77–07/88 (*n* = 248)	08/88–02/00 (*n* = 454)	
Age at primary episode (years) (mean ± DP/median)	8.8 ± 5.5/8.8	9.4 ± 3.1/9.0	0.0823		
Gender (F/M)	1.2	1.01	0.9090	1.041	0.598–1.812
Large/small joints (%)	86.7/13.3	84.5/15.3	0.3548	1.084	0.744–1.580
ΣMR, MR+AR, AR+MR+MS, MR+MS /other associations (%)	94.2/5.8	95.6/4.4	0.3637	1.532	0.419–5.603

*MS, mitral stenosis; MR, mitral regurgitation; AR, aortic regurgitation*.

**Table 4:** **Comparative analysis of frequency of recurrences and indices of severity**.

Indices of severity	Time interval		*p*	OR	CI
	07/77–07/88 (*n* = 248)	08/88–02/00 (*n* = 454)	
Recurrences	22.4	7.4	0.0000	2.163	1.222–4.169
Severe/mild carditis	49.8/19.6	30.1/43.2	0.0000	1.556	1.129–2.144
Frequency of MS*/MR and/or AR	33.2/66.8	11.9/88.1	0.0000	2.129	1.287–3.523
Hospitalization	45.4	28.4	0.0000	1.478	1.065–2.052
Surgeries	13.3	1.5	0.0000	3.973	1.860–14.539
Deaths	5.4	0.4	0.0000	3.591	1.582–22.156

MS, mitral stenosis; MR, mitral regurgitation; AR, aortic regurgitation, *associated with MR and/or AR.

## Discussion

The present study confirms previous results of frequent involvement of the heart in children and adolescents with RF and the influence of recurrences in the severity of valvular sequels (5). The more patients are faced with recurrent episodes, the more advanced disease and its complications can be anticipated in the chronic phase (4). This poor outcome was seen prior to the implementation of prophylactic measures and was represented by a higher demand for hospitalization and surgery besides a higher number of deaths. Similarly, a prevalence of mitral stenosis three times higher than that presented after the control of recurrences was found, showing a shorter and unexpected time course for this age group. This accelerated progression into severe forms contrasts with the most often latent period of 20–40 years between the presentation of the obstructive lesion and the episode of RF. This trend has already been reported and may reflect the final result of multiple insults (17, 22). At the other end of the spectrum, we highlight the potential regression of less significant valvular regurgitation. The involution of valvular disease at echocardiographic interrogation observed in 34.3% of children and adolescents in the present investigation reinforces the need for a timely identification of patients at an early stage of the illness. Patients with mild valvular dysfunction and submitted to regular secondary prophylaxis have shown a good prognosis without detectable disease after 10 years (23, 24). A better cardiac condition at the time of the initial attack has also been pointed out as a factor related to improvement of mitral regurgitation (25). In this setting, the routine echocardiographic screening for all patients included in the program, even for suspect cases without clinical evidence of valvar dysfunction, contributed to an early identification of mild dysfunctions, clinical or subclinical.

As expected, significant degrees of carditis were more frequently seen in patients during subsequent attacks when compared with those at primary episodes. In the comparative analysis, the control of recurrences was followed by a decrease in the frequency of severe carditis from 49.8 to 30.1% and an increase of mild carditis from 19.6 to 43.2%. Additionally, the prevalence of severe cardiac involvement observed after the decrease of recurrences (30.1%) was similar to the percentual found in all patients at a primary attack (30.3%). These findings can be related to a timely diagnosis and the more effective measures to prevent the progression of the cardiac damage mainly in patients at the initial episode, where less significant degrees of carditis are expected.

In countries such Brazil, programs to implement the delivery of secondary prophylaxis have been recommended as a priority for RHD control (26, 27). This approach has shown to be cost-effective at population and community levels (26–28). When compared to primary prevention, this RHD control strategy is targeted at a small and specific populational group, and can be integrated into the existing healthcare system. It has been estimated that programs to improve secondary prophylaxis cost less than half the amount spent on tertiary prevention and less than one-seventh of that spent on primary prevention (29). The control program at UFMG has required a multi-faceted approach including continuous surveillance, and according to our observations, no single measure could be exclusively related to the outcomes. Previous divulgation by the media and the incorporation of activities into the existing health system infrastructure supported the training of healthcare providers, which was followed by the establishment of the Reference Center and the implementation of activities. Appropriate delivery of reliable forms of benzathine penicillin and the development of strategies to promote the adherence to prophylactic regimens were crucial to reach our goals. In this setting, we highlight the introduction of the prophylaxis card to control the compliance of patients and the combined actions of the multidisciplinary health team in providing information and improving the clinical assistance and relationship with patients and their families.

Considering the necessity of a continuous surveillance, the better adherence to a regular follow-up allowed the monitoring of the clinical evolution at least twice a year. It also contributed to reinforce the commitment to the use of medication besides improving communication with the referring healthcare centers. Special attention was given to adolescents. This age group was less likely to show adequate compliance to penicillin injections. As regards the ongoing debate about the interval between the doses of intramuscular benzathine penicillin for the implementation of the secondary prophylaxis, whether it should happen every 3 or 4 weeks (24, 30), our view is that the approach must be tailored to the epidemiological context. Our choice of the three weekly regimens took into account the frequency of the disease and the individual risks of exposure to group A streptococcu*s*.

Although important modifications in the morbimortality indices were found in the present investigation and had already been observed in other RHD control programs, there is a clear necessity of increasing our understanding of this preventable and baffling disease, which continues to challenge clinicians and the scientific community. Moving forward, efforts must be directed toward research. In prospect, our more promising advances include the availability of a streptococcal vaccine for an effective access to primary prevention (31). As it has been pointed out by Carapetis and Zühlke in the discussion about the priorities for the global research in RF and RHD, “the major challenge, however, is not to miss the very real opportunity that now exists to translate current and future research into concrete action” (32). As a result, informed decision can be made and strategies can be defined for intervention at the individual and public health levels.

In conclusion, the results of this investigation showed that the cardiac involvement in RF remains frequent as does the association of recurrences with significant valvar dysfunction. In the scenario of the substantial burden imposed by the disease and poor long-term outcomes, the implementation of a program of secondary prophylaxis is an effective strategy to prevent recurrent RF and progression of RHD.

### Limitations of the study

This study has limitations as the data were collected retrospectively and the investigated databases and medical reports cannot be proved to be either complete or comprehensive. In addition, the quality of the Doppler echocardiographic approach and consequently the diagnosis accuracy of valvar lesions were not linear as technology has improved throughout the long period of investigation as regards the methods and quality of the equipment.

## Conflict of Interest Statement

The authors declare that the research was conducted in the absence of any commercial or financial relationships that could be construed as a potential conflict of interest.
